# Sonographic features of the testicular adrenal rests tumors in patients with congenital adrenal hyperplasia: a single-center experience and literature review

**DOI:** 10.1186/s13023-019-1231-1

**Published:** 2019-11-06

**Authors:** Li Ma, Yu Xia, Linlin Wang, Ruifeng Liu, Xuepei Huang, Tiantian Ye, Li Zhang, Qingli Zhu, Jianchu Li, Yuxin Jiang

**Affiliations:** 1 Department of Ultrasound, Peking Union Medical College Hospital, Chinese Academy of Medical Sciences, Shuaifuyuan 1, Dongcheng District, Beijing, 100730 China; 2 Department of Ultrasound, Harrison International Peace Hospital, Hebei, China

**Keywords:** Testicular adrenal rests tumor, Ultrasound, Congenital adrenal hyperplasia

## Abstract

**Purpose:**

Testicular adrenal rests tumor (TART) is a rare kind of benign tumor in the testis. It usually occurred secondary to congenital adrenal hyperplasia (CAH), a hormonal disorder caused by hydroxylase deficiency. As the first-line examination method, ultrasound provides crucial diagnostic information for TART, although misdiagnosis to malignancy is quite common because of its rare prevalence. We aimed to summarize the sonographic manifestations of TART to improve the diagnostic accuracy and specificity.

**Methods:**

Eight CAH patients with TART identified by ultrasound in our medical center were retrospectively reviewed. Clinical and hormonal profile, semen analysis and treatment choices were collected. Sonographic examinations were performed at the first evaluation and interpreted by experienced radiologists individually. All patients received regular follow-up, and 5 patients undertook repeated scrotal ultrasound. A literature review of TART in CAH patients was conducted, with 123 patients from 23 articles since 1990 included.

**Results:**

A total of 8 patients aged between 4 to 27 years old were enrolled. 7 of 8 (87.5%) patients exhibited bilateral testicular lesions. The sizes of the testicular lesions were between 0.18 ml to 5.68 ml, and all showed a clear boundary. 10/15 (66.7%) lesions were homogenously hypoechoic, 4/15 (26.7%) were heterogeneously iso-hypoechoic, and 1/15 (6.7%) were homogenously isoechoic. 10/15 (66.7%) lesions were hyper-vascular. The longitudinal follow-up of 5 patients showed testicular lesions changed in terms of size, echogenicity, and vascularity after steroid treatment. A potential correlation may exist between ACTH levels and tumor size (*p* = 0.066). From the literature review, 100/123 (81%) patients got bilateral lesions, and 95% of them were located near the mediastinum. 80/103 (78%) lesions exhibited a clear boundary, and predominant lesions (74%) were hypoechogenic. Vascularity was with great diversity. Seventy-nine lesions of 44 patients were followed-up by scrotal ultrasound, among which 29 (37%) remained unchanged, 29(37%) shrank, and 21(27%) disappeared.

**Conclusions:**

Key sonographic characteristics of TART are: resembled lesions on both testes, located near the mediastinum, clear boundary, and changed in size or echogenicity after steroid treatment. These features can help radiologists to make an accurate diagnosis of TART.

## Introduction

Testicular adrenal rests tumor (TART) is a rare kind of benign tumor in the testis, which occurs mainly secondary to congenital adrenal hyperplasia (CAH), an autosomal recessive disorder with a deficit of enzymes related to glucocorticoids synthesis. In over 90% cases, the deficient enzyme is 21-hydroxylase, while in other cases, the deficient enzyme is 11-hydroxylase [1]. Driven by the negative feedback regulation, the level of adrenocorticotropic hormone (ACTH) increases, leading to the hypertrophy of the adrenal glands, and overproduction of other hormones [2]. During embryonic development, some cells destined to become adrenal glands may nestle within the rete testis because of the close positional relation, known as residual adrenal cells [3]. The reported prevalence of residual adrenal cells in the testes of healthy neonates was 15%, although it is probably underestimated due to technical reasons [4, 5]. Nowadays, it is believed that TART originates from these residual adrenal cells, and high ACTH levels stimulate these cells to proliferate and form masses. The prevalence of TART in CAH male patients varies considerably in previous reports, with an increasing prevalence rate observed during and after puberty [6, 7]. Although it is benign, the growing masses would compress the spermatogonium and ductulus efferens, which may lead to irreversible damage and cause infertility [8, 9]. Ectogenous glucocorticoids are used for CAH and TART treatment by suppressing ACTH production and maintaining the glucocorticoid level [10].

As a safe, convenient, and economic-efficient method, scrotal ultrasound is usually recognized as the first-choice for TART screening in clinical practice. It is indicated that ultrasound and MRI have comparable sensitivity in the detection of TARTs [11]. Caused by its low prevalence, only limited literature has reported the sonographic features of TART; most radiologists do not recognize it and misdiagnose it to malignant tumors, which may lead to unnecessary testectomy [12]. Here, to give a thorough sonographic description of TART, we presented 8 cases of TART in our hospital, and conducted a literature review of the previous reports.

## Methods

### Patients

This is a retrospectively study, where the inpatient and outpatient databases from January 2004 to December 2017 in Peking Union Medical College Hospital were reviewed to identify patients diagnosed with congenital adrenal hyperplasia. In all patients, a diagnosis of 21-hydroxylase deficiency or 11β-hydroxylase deficiency was confirmed by DNA analysis. Scrotal ultrasound must be performed at the first evaluation. TART was diagnosed by either pathological examination or clinical diagnostic criteria that testicular lesions had a good response to hormonal therapy. The demographical, clinical, hormonal, radiological, and pathological data of TART patients before and after treatment were collected. Patients were treated with either dexamethasone or hydrocortisone, and they were asked to come back after treatment for 6 months, when repeated hormonal and/or ultrasound examinations were performed. Written informed consent was signed by all patients or their legally authorized representatives with regard to the use of patients’ data for study propose.

### Semen analysis

Samples of sperm were collected by masturbation after 3 to 5 days of sexual abstinence. Sperm concentration and motility were assessed by a superimposed image analysis system, in accordance with the World Health Organization recommendations [13]. According to WHO recommendations, aspermia was defined as the inability to deliver semen, and azoospermia defined as the absence of spermatozoa in the ejaculate [13].

### Ultrasound examination

Scrotal ultrasound examinations were performed to all CAH male patients before hormone therapy started. Five patients had performed follow-up scrotal ultrasound after steroid therapy started. The ultrasound machine is iU22 machine (Philips Healthcare, Amsterdam, Netherland) equipped with a transducer with a frequency range of 5–12 MHz. All ultrasound examinations were performed by one radiologist with more than 5 years of experience in ultrasound. Both gray-scale and color Doppler images were saved. Then the images were interpreted by another two radiologists specialized in testicular ultrasound individually, and the number, size, shape, boundary, echogenicity, and vascularity of the testicular lesions were carefully recorded in written reports. Lesion shape was divided into “round” “lobular” and “irregular”, with a round shape defined as spherical or elliptical, a lobular shape defined as undulating contour, and an irregular shape defined as uneven shape (not round or lobular) [14].“Clear boundary” was defined as the lesion margins that can be clearly defined. The echogenicity was basically divided into hypoechogenicity, isoechogenicity, and hyperechogenicity, as compared to the echogenicity of the normal testis. The echogenicity of the lesion was divided into “homogeneous” and “heterogeneous”. “Heterogeneously iso-hypoechoic” is defined as the lesion that is heterogeneous with both isoechogenicity and hypoechogenecity. The evaluation of vascularity was graded according to the Adler’s method, that the vascularity was subjectively determined to be absent (grade 0), minimal (grade 1), moderate (grade 2), or marked (grade 3) with reference to the normal region of testis, which was considered as moderate vascularity [15].

### Literature review

A thorough literature search was conducted in MEDLINE and Embase databases. The keywords we used for literature search is “testicular adrenal rest tumors ultrasound” within the time range from January 1990 to June 2019. There were 86 results in Pubmed and 150 results in Embase. The inclusion criteria were: [1] Confirmed diagnosis of TART [2]; scrotal ultrasound was performed, and ultrasonographic features were detailedly described (at least lesion size and echogenicity). The excluded criteria were: [1] reviews, irrelevant or overlapped articles [2]; articles that were not able to get access to the full text. Finally, 23 articles were included. Relevant literature was carefully read and compared to our observation.

### Statistics

The correlation of the size of TART and levels of ACTH, luteinizing hormone (LH), follicle-stimulating hormone (FSH), testosterone and age was estimated by Pearson Correlation Coeffient. Lesion size was calculated by the formula: ellipsoid volume = π × length×width×height÷6. Bilateral tumors in the same patient were regarded as two individual tumors.

## Results

### Baseline characteristics

In our databases, 48 male patients were identified to be diagnosed as CAH, and all of them underwent scrotal ultrasound examinations. Testicular masses were found by ultrasound in 8 patients, and all of them were diagnosed with TART, among whom three by pathologically confirmed as the residual adrenal gland tissue, and five by meeting the clinical diagnostic criteria that testicular lesions had a good response to hormonal therapy. The baseline characteristics of 8 TART patients were summarized in Table 1. Eight patients were aged from 4 to 27 (median age, 16), with the height from 119 to 169 cm (median height, 155.7 cm) and the weight from 24 to 74 kg (median weight, 59.1 kg). CAH diagnosis was made between 0 and 13 years old. The majority (7/8) of patients was caused by 21-α-hydroxylase deficiency, while only one patient was caused by 11-β-hydroxylase deficiency. Blood pressures of all patients were within the normal range (from 95/65 to 120/78 mmHg). Six of 8 TART patients undertook semen analysis. From semen analysis, one 24-year-old patient suffered from aspermia, another 27-year-old patient had azoospermia, and four patients showed normal results. Among andrological examinations, 7 of 8 (87.5%) patients had palpable testis lesions, 1 of 8 (12.5%) had the varicocele, and no patients showed epididymal cyst and gynecomastia. The hormonal levels showed a broad distribution (Table 2). Five patients with HCG and AFP records showed normal levels of both markers.

**Table 1:** Baseline characteristics of TART patients

Patient No.	Age at CAH diagnosis (years)	Age at TART diagnosis (years)	Time between CAH and TART diagnosis (years)	CAH Type	Ht (cm)	Wt (kg)	Blood Pressure (mmHg)	Semen analysis		Andrological examinations			
								Aspermia	Azoospermia	Palpable testis nodules	Vari-cocel-e	Epididy-mal cyst	Gynaecom-astia
1	6	27	21	21-α-hydroxylase deficiency	169	74	120/70	Yes	No	Yes	No	No	No
2	4	24	20	21-α-hydroxylase deficiency	160	60	120/70	No	Yes	Yes	Yes	No	No
3	0^a^	18	18	21-α-hydroxylase deficiency	154	64	120/70	No	No	Yes	No	No	No
4	13	16	3	21-α-hydroxylase deficiency	158	72	110/78	No	No	Yes	No	No	No
5	0	15	15	11-β-hydroxylase deficiency	162	46	100/60	No	No	Yes	No	No	No
6	2	16	14	21-α-hydroxylase deficiency	163	50	95/60	No	No	Yes	No	No	No
7	0	13	13	21-α-hydroxylase deficiency	158	73	100/60	/	/	Yes	No	No	No
8	0	4	4	21-α-hydroxylase deficiency	118	24	95/65	/	/	No	No	No	No

Abbreviations: *Ht* height, *Wt* weight

^a^0 means that CAH was diagnosed before 1 year old

**Table 2:** Hormone and biomarker levels of TART patients before and after treatment

Patient No.	Treatment	LH (IU/L)		FSH (IU/L)		PRL (ng/mL)		P (ng/mL)		T (ng/mL)		E (pg/mL)		ACTH(pg/ml)		17-OHP(ng/ml)		HCG	AFP
		Pre	Post	Pre	Post	Pre	Post	Pre	Post	Pre	Post	Pre	Post	Pre	Post	Pre	Post		
1	Dexamethasone	5.1	8.21	2.96	4.44	12.6	/	10.94	33.06	553.4	7.17	20	38	282	/	29.5	/	<5	2.4
2	Hydrocortisone	20.1	/	0.5	/	/	/	20	/	18.7	/	99.48	/	157	/	299.9	/	<0.5	1.9
3	Dexamethasone	14.34	12.79	17.29	24.95	/	/	5.44	2.85	1.71	2.46	19	12	539	41.2	84.5	3.92	/	/
4	Hydrocortisone	6.41	0	6.42	0	7.24	35	/	/	25.51	3.66	38	240.6	32.4	14.6	40.1	19.68	0	1.7
5	Hydrocortisone	4.44	8.59	8.21	10.45	/	/	33.06	5.27	2.17	6.23	38	27	410	516	DS: 184.6	DS: 200.6	/	/
6	Hydrocortisone	2.66	11.38	6.3	6.22	/	/	15.3	6.68	5.54	6.29	27	23	291	798	DS: 89.3	DS: 74.4	/	/
7	Prednisone	0.96	1.88	5.21	5.49	/	/	2.7	4.88	3.59	4.36	5	35	254	213	DS: 29.0	DS: 36.1	<0.1	1.7
8	Hydrocortisone	0.18	1.10	0.76	0.21	/	/	39	0.39	2.07	< 0.1	18	< 5	373	170	64.31	4.47	0.8	1.1

Abbreviations: *LH* luteinizing hormone, *FSH* follicle-stimulating hormone, *PRL* prolactin, *P* progesterone, *T* testosterone, *E* estrogen, *ACTH* adrenocorticotropic hormone, *17-OHP* 17 hydroxyprogesterone, *HCG* human chorionic gonadotropin, *AFP* alpha fetoprotein, *Pre* pre-treatment, *Post* post-treatment, *DS* dehydroepiandrosterone-s

### Ultrasound manifestations

From ultrasound results, seven patients had bilateral lesions, whereas one patient had a unilateral lesion; therefore, the total number of lesions is 15 (Table 3). All lesions were located near the testicular mediastinum. The size of the 15 lesions ranged from 0.18 ml to 5.68 ml. The *p*-value between tumor size and ACTH levels was 0.066. No statistically significant correlation of tumor size and age was found (*p* = 0.328), neither did LH (*p* = 0.285), FSH (*p* = 0.947) and testosterone (*p* = 0.659). Same sonographic manifestations were found in bilateral lesions of the same patient. The shapes of the lesions were round (9 lesions) or lobular (6 lesions). All lesions had a clear boundary. Ten lesions were homogeneously hypoechoic, four were heterogeneously iso-hypoechoic, and one was homogeneously isoechoic. The majority of lesions had marked vascularity (in 10 lesions), while four lesions had minimal vascularity, one showed an absence of vascularity. Calcification was found in two lesions of the same patient.

**Table 3:** Sonographic features of testicular adrenal rests tumor in TART patients

Patient No.	Lesions	Lesion size (ml)	Lesion Shape	Lesion Boundaries	Echogenicity	Vascularity	Calcification
1	L	0.21	Round	Clear	Homo/iso*	Absent	No
2	L	2.09	Round	Clear	Homo/hypo	Marked	No
	R	1.52	Round	Clear	Homo/hypo	Marked	No
3	L	5.13	Round	Clear	Hetero/iso-hypo	Minimal	No
	R	5.68	Round	Clear	Hetero/iso-hypo	Minimal	No
4	L	0.30	Lobular	Clear	Homo/hypo	Marked	Yes
	R	0.20	Lobular	Clear	Homo/hypo	Marked	Yes
5	L	0.34	Round	Clear	Homo/hypo	Marked	No
	R	0.18	Round	Clear	Homo/hypo	Marked	No
6	L	1.48	Lobular	Clear	Hetero/iso-hypo	Marked	No
	R	1.83	Lobular	Clear	Hetero/iso-hypo	Marked	No
7	L	0.90	Lobular	Clear	Homo/hypo	Marked	No
	R	0.92	Lobular	Clear	Homo/hypo	Marked	No
8	L	1.49	Round	Clear	Homo/hypo	Minimal	No
	R	0.47	Round	Clear	Homo/hypo	Minimal	No

Abbreviations: *L* left, *R* right, *homo* homogeneous, *iso* isoechogenicity, *hypo* hypoechogenicity, *hetero* heterogeneous, *iso-hypo* hypoechogenicity

mixed with isoechogenicity

**Fig. 1:**
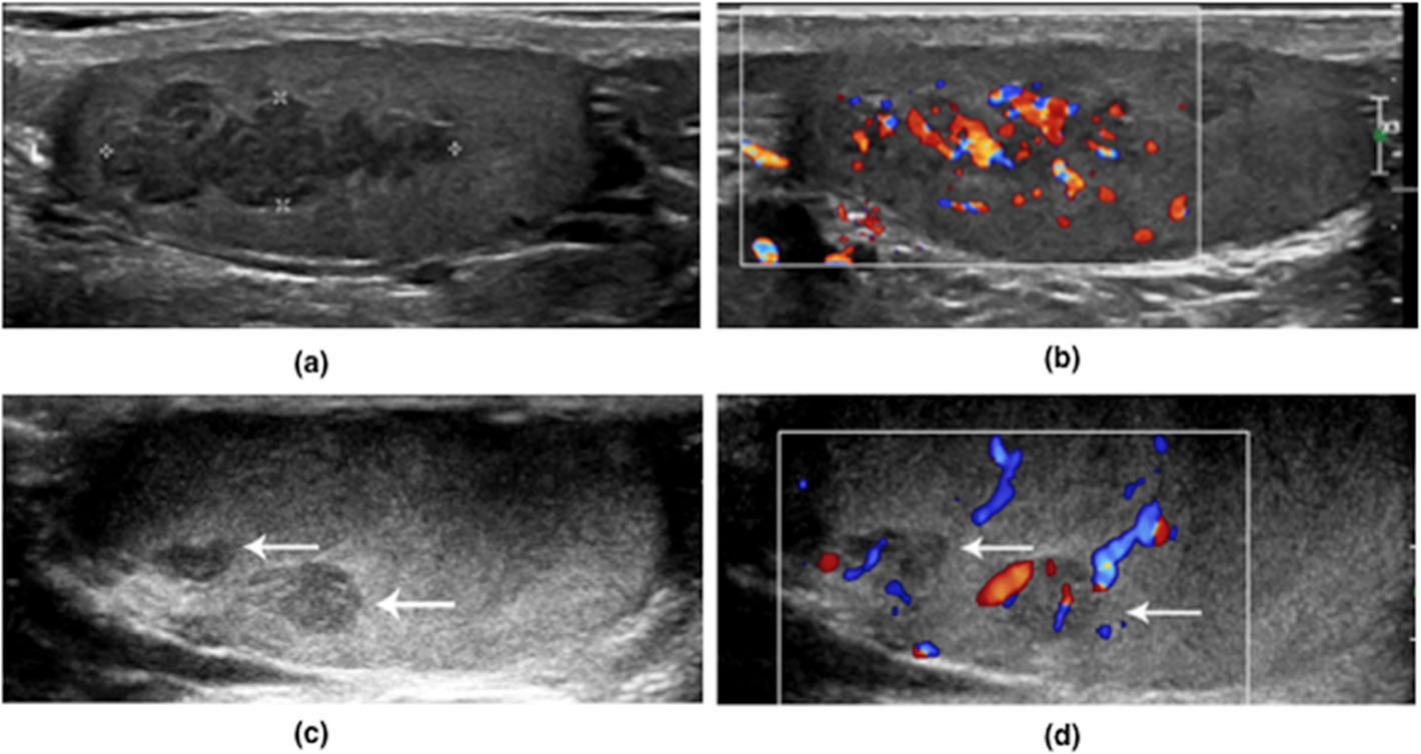
Ultrasound features of TART. This is a 13-year-old CAH patient. Palpable testicular nodules were detected through physical examination and a scrotal ultrasound was therefore performed. The scrotal gray-scale ultrasound (**a**) and color Doppler ultrasound (**b**) showed homogeneous hypoechoic lesions with clear boundary on both testes with marked vascularity. Follow-up gray-scale (**c**) and color Doppler (**d**) ultrasound examination was performed after a 6-month steroid treatment, which showed a remarkable decrease in lesion size and vascularity

Five patients received follow-up scrotal ultrasound 6 months after steroid treatment. One patient had a poor hormonal control during the treatment, and sonographic images showed a similar lesion size as before, but a change from the round shape from to lobular shape. Four patients had good hormonal control during the treatment, and testicular lesions reduced significantly in the size and vascularity, without prominent alterations in the shape, boundary, and echogenicity (Fig. 1).

### Literature review

In the literature since 1990, 23 articles were found to have described the sonographic features of TART [2, 7, 11, 16–34]. One hundred twenty-three patients with a total of 223 testicular lesions were collected. Results were presented in Table 4. 100 (81%) patients had bilateral lesions, and the majority of them were located near the mediastinum (95%). Most lesions were round (oval) (75/106, 71%) or lobular (29/106, 27%). 80/103 (74%) lesions had a clear boundary; 164 (74%) lesions were hypoechoic, 41 (18%) were heterogeneous, and 18 (8%) were with other types, such as hyperechoic. As for vascularity, 39/152 (26%) lesions exhibited an absence of blood signal, 25/152 (16%) exhibited a minimal blood supply, 31/152 (20%) had a moderate blood supply, and 52/152 (34%) exhibited a marked blood supply. Lastly, 79 lesions of 44 patients were followed-up after steroid therapy, among which 29 (37%) lesions remained unchanged, 29 (37%) shrank, and 21 (27%) disappeared.

**Table 4:** Literature review of testicular adrenal rests tumor in patients with congenital adrenal hyperplasia

Characteristics		Number (Percentage)
Total number of patients/lesions		123/223
Bilateral lesions		100/123 (81%)
Located near the mediastinum		107/113 (95%)
Clear Boundaries		80/103 (78%)
Shape	Round or oval	75/106 (71%)
	Lobular	29/106 (27%)
	Irregular	2/106 (2%)
Echogenicity	Hypoechogenicity	164/223 (74%)
	Heterogeneous	41/223 (18%)
	Other types^a^	18/223 (8%)
Vascularity	Absent	39/152 (26%)
	Minimal	25/152 (16%)
	Moderate	31/152 (20%)
	Marked	52/152 (34%)
Follow-up	No change	29/79 (37%)
	Reduced	29/79 (37%)
	Disappear	21/79 (27%)

^a^Other types: hyperechogenicity, isoechogenicity, or anechogenicity

## Discussion

In this study, we retrospectively reviewed the sonographic characteristics of TART in 8 CAH patients, and further analyzed 123 CAH patients from the literature. The TARTs were described mainly in several aspects: bilateral/unilateral; location; size; shape; boundary; vascularity; changes with steroid treatment. The sizes of lesions from the literature were not summarized this time because of the nonuniform descriptions. We also described the calcification of two lesions in one patient, suggesting that calcification is not an exclusive sign for malignant tumors.

The majority of TART are bilateral. Notably, we also noticed that the same characteristics were observed in bilateral lesions in every patient, including echogenicity, boundary, and vascularity. This feature is also confirmed by several other studies, for example, Wang et al. reported 15 TART patients, and 12 of 15(80%) showed exact same sonographic features of both lesions [21]; Defino et al. described 9 bilateral TARTs with same characteristics [2]; Multiple case reports also described this feature [16, 19]. As most malignant tumors occur solitarily, it is a distinct feature for differential diagnosis. However, it is worth noticing that unilateral lesions cannot rule out TART. The reason is probably that minimal lesions may exist but are undetectable by radiological methods. Similarly, although the clear boundary is a prominent feature of TART, it is not specific enough, since other kinds of testicular tumors like seminoma, teratoma, Leydig cell tumor, can also have clear boundaries on the ultrasound.

Controversial opinions exist about the relationship between ACTH levels and the size of TARTs [7]. Some studies reported that TART cells have ACTH receptors so that the tumor size is responsive to ACTH levels, while other studies suggested that other growth-promoting factors might also be involved, thereby ACTH is not the only factor effecting on the growth of TART [6, 8, 35]. In our study, a *p*-value of 0.066 was found between TART size and ACTH levels, suggesting a potential relationship may exist, even though further studies with a larger sample size are needed for the validation of our results.

In our study, most lesions showed marked blood supply. Summarized from the literature, the vascularity of TART exhibited a great diversity. Interestingly, different researches revealed completely opposite vascularity conditions, for example, in the report of Wang et al., the majority of lesions were richly vascularized, whereas in the study of Delfino et al., no blood signal was observed in a large percentage of lesions [2, 21]. Since the vascularity assessment lacks objective values such as peak systolic velocity or resistance index in this condition, it depends mainly on the different machine settings and subjective judgment by radiologists, making it an unreliable indicator. Further studies are encouraged to provide objective parameters of vascularity for clinical use. In this circumstance, vascularity cannot become a suitable marker for diagnosis and differential diagnosis.

From the literature, shrinkage or disappearance of TARTs was observed in 64% (50/79) lesions after steroid therapy. A few patients failed to show any changes during follow-up, and the potential reason might be poor steroid control with/without treatment. Ultrasound can be an ideal tool for TART monitoring. Previous studies have proposed that an ultrasound screening for TART should be performed in all CAH patients, and regular monitoring by scrotal ultrasound should be done to prevent possible infertility [36–38].

Besides sonographic features, TART always accompanies with prominent clinical manifestations related to CAH, such as obesity, short stature, early hypertension, and hirsutism [8, 39]. These clinical characteristics are easily perceived when performing ultrasound examinations or accessed by enquiring patients or their guardians. Identification of these features might increase the diagnostic confidence of TART. Normal AFP and HCG levels are also suggestive of benign tumors. However, since other testicular tumors such as Leydig cell tumors can also lead to precocious puberty in pre-pubertal males, radiologists would be very careful to make a differential diagnosis and not rely too much on clinical features [40]. Sonographically, Leydig cell tumors are mostly isolated, hypoechoic masses with peripheral vascularity [41].

There are several limitations to our study. The sensitivity and specificity of each characteristic sonographic sign were not able to be calculated; therefore, a more thorough case-control study with a larger sample size is necessary for the future. How the hormones affect TART evolvement was not able to be addressed in this study.

## Conclusions

In conclusion, according to our findings, TART has several prominent features on ultrasound: [1] bilateral testicular lesions with the same manifestations [2]; located near the mediastinum [3]; clear boundary [4]; sonographic changes with steroid therapy. As a rare kind of benign tumor in the testis, TART has to be diagnosed very carefully, and a reference to typical CAH symptoms, AFP, HCG, and hormonal levels and a positive finding in adrenal glands can be of help to increase diagnostic accuracy.

## Data Availability

The data and material can be provided if asked on a basis of good reasons.

## Abbreviations

ACTH: adrenocorticotropic hormone
CAH: congenital adrenal hyperplasia
FSH: follicle-stimulating hormone
LH: luteinizing hormone
TART: testicular adrenal rests tumor
